# Motor Imagery Performance and Tactile Spatial Acuity: Are They Altered in People with Frozen Shoulder?

**DOI:** 10.3390/ijerph17207464

**Published:** 2020-10-14

**Authors:** John D. Breckenridge, James H. McAuley, Karen A. Ginn

**Affiliations:** 1 School of Medical Sciences, Faculty of Medicine and Health, The University of Sydney, Sydney 2006, Australia; karen.ginn@sydney.edu.au; 2 The Clinical Research Institute, Sydney 2145, Australia; 3 Neuroscience Research Australia (NeuRA), Hospital Rd, Randwick 2013, Australia; j.mcauley@neura.edu.au; 4 School of Medical Sciences, Faculty of Medicine, University of New South Wales, Sydney 2033, Australia

**Keywords:** left/right judgement, two-point discrimination, adhesive capsulitis, chronic pain

## Abstract

Frozen shoulder (adhesive capsulitis) is a severe chronic pain condition that is not well understood and current treatment is suboptimal. In several other chronic pain conditions motor imagery and tactile acuity deficits are present, which are thought to represent associated neuroplastic changes. The aims of this study were to determine if motor imagery performance assessed by the left/right judgement task, and tactile acuity assessed by two-point discrimination, are altered in people with unilateral frozen shoulder. In this cross-sectional, prospective study eighteen adults diagnosed with frozen shoulder in a physiotherapy clinic setting completed a left/right judgement task, response times (RT) and accuracy for the left/right judgement task were determined. Next, tactile acuity over both shoulders was assessed with a novel, force-standardised two-point discrimination test. Results corresponding to the affected side were compared to the pain free shoulder; Left/right judgement task: mean RT (SD) corresponding to the affected shoulder was significantly slower than RT for the healthy shoulder (*p* = 0.031). There was no side-to-side difference in accuracy (*p* > 0.05). Neither RT nor accuracy was related to pain/disability scores or duration of symptoms (*p* > 0.05). Two-point discrimination: mean two-point discrimination threshold of the affected shoulder was significantly larger than the contralateral healthy shoulder (*p* < 0.001). Two-point discrimination threshold was not related to pain/disability scores or pain duration (*p* > 0.05); One explanation for these findings is altered sensorimotor processing and/or disrupted sensorimotor cortex representations of the affected shoulder. A case then exists for the use of treatments aimed at reversing these changes, training the brain to reduce chronic shoulder pain.

## 1. Introduction

Frozen shoulder (adhesive capsulitis) is a severe chronic musculoskeletal pain condition, with a typical history of insidious onset shoulder pain, married with progressive stiffness and functional limitation [1] lasting for many months. The natural history of frozen shoulder is commonly reported to consist of three overlapping stages: a progressively painful stiffening ‘freezing’ stage; a ‘frozen’ stage of extreme range of motion restriction; and a ‘thawing’ stage as stiffness resolves [2]. However, this description of the course of frozen shoulder has been disputed in a recent systematic review [3], and current evidence based physiotherapy guidelines recommend classifying frozen shoulder as ‘pain predominant’ or ‘stiffness predominant’ [4]. Frozen shoulder has been associated with metabolic diseases—diabetes mellitus [5], hyperthyroidism [6]—and chronic musculoskeletal conditions e.g., Dupuytren’s disease [7] and complex regional pain syndrome [8]. Suggested causal factors include contracture of capsular structures, inflammatory agents, collagen changes, neovascularization [9] and muscle guarding [10,11], however, the pathophysiology of frozen shoulder is unknown [12]. There is no definitive diagnostic test for frozen shoulder [13,14] which is determined by a clinical examination confirming restriction of active and passive shoulder range of motion, normal glenohumeral radiograph, and exclusion of other shoulder pathologies. 

Frozen shoulder is perhaps the least understood of shoulder pathologies [1] and a century after Codman first coined the term frozen shoulder it remains “difficult to define, difficult to treat and difficult to explain” [15]. Recommendations for treatment of frozen shoulder are suboptimal and conflicting ranging from ‘benign neglect’ [16], to mobilisation techniques [17], corticosteroid injection and/or exercise [1,18]. The natural history tenets that frozen shoulder is self-limiting and full resolution without supervised treatment can be expected is not supported by systematic review evidence [3]. Given that frozen shoulder has proven resistant to treatment, exploration of emerging treatments is warranted.

Brain abnormalities [19,20] have been associated with a number of chronic pain conditions, and include alterations in S1 representations of the painful body part in chronic back pain [21] and complex regional pain syndrome [22], and motor cortical changes in knee osteoarthritis [23], persistent elbow pain [24], rotator cuff tendinopathy [25] and complex regional pain syndrome [26]. Perceptual and proprioceptive alterations in back/neck pain [27,28,29,30,31], knee osteoarthritis [32,33,34], complex regional pain syndrome [35,36,37] and shoulder pain [38] suggest central abnormalities. Additionally, tactile acuity impairments in a variety of chronic musculoskeletal pain conditions are thought to reflect functional reorganisation of the somatosensory cortex [39]. It is unknown whether these central changes are a feature of frozen shoulder.

Pain related cortical changes can be explored indirectly by motor imagery, one method being the left/right judgement task (LRJT) [40] requiring activation of proprioceptive, somatosensory, premotor and related regions. Altered motor imagery performance seems to be a feature of a number of chronic pain conditions [41], but there is uncertainty whether motor imagery performance is altered in people with chronic shoulder pain [42,43]. A second method of exploring pain related somatosensory changes is through tactile acuity testing and the prevailing approach is the two-point discrimination threshold test [44]. Tactile acuity deficits have been described in a variety of chronic pain conditions, including a recent study in people with frozen shoulder [42], indicating somatosensory cortex reorganisation in these patients [39]. 

In other chronic musculoskeletal pain conditions treatment directed at normalising cortical changes have demonstrated some success. Graded motor imagery [45,46] which aims to normalise proprioceptive and cortical motor networks has shown positive outcomes in the treatment of complex regional pain syndrome [45,47] and phantom limb pain [48]. Sensory discrimination training has demonstrated reduction in both phantom limb pain and cortical reorganisation in amputees [49], reduced pain in complex regional pain syndrome [50] and may improve pain and function in chronic low back pain [51]. An approach combining graded motor imagery and sensory discrimination training has shown promise in a chronic low back pain case series [52] and a frozen shoulder case study [53]. If cortical changes are associated with frozen shoulder, then graded motor imagery and tactile acuity training may prove to be helpful in the treatment of this condition for which effective treatment remains elusive.

The primary aims of this cross-sectional study were to examine if motor imagery performance and tactile acuity are altered in people with unilateral frozen shoulder, implicating cortical changes. We hypothesised that performance on the shoulder LRJT and two-point discrimination threshold, would differ between the affected and healthy shoulders. Potential relationships were explored between LRJT/ two-point discrimination outcome measures and pain levels, functional limitation, and age.

## 2. Materials and Methods

### 2.1. Ethics

All study procedures were approved by the University of Sydney Human Ethics Committee (Protocol No. 05-2011/13645, Project reference number 2012/539).

### 2.2. Participants

Participants diagnosed with frozen shoulder, were recruited from four physiotherapy private practices in Western Sydney, NSW, Australia from August 2015 to December 2017. Adults presenting with a unilateral, painful, stiff shoulder, of unknown cause, for at least six weeks duration, were eligible for this study. Participants were required to have at least one third restriction of active/passive shoulder range of motion, in at least two planes. Participants were excluded if they had evidence of osteoarthrosis on a shoulder radiograph, inflammatory joint disease, referred pain past the elbow or paraesthesia indicating neurological changes.

Frozen shoulder participants read the patient information statement provided and signed a consent form. Participant data collected included: age, gender, affected shoulder side and duration of frozen shoulder symptoms. Participants completed two numerical pain scales (NRS) [54], one for worst shoulder pain, and another for average shoulder pain over the previous week, and the Shoulder Pain and Disability Index (SPADI) [55,56]. Both active and passive shoulder range of motion were recorded for the affected and unaffected shoulder [57]. This included shoulder flexion and external rotation measured using a standard goniometer, and hand-behind-back measured by recording the anatomical landmark reached by the thumb tip. Additionally, light touch sensory profile was assessed over both shoulders to check for areas of numbness by repeatedly placing a 4.08 Semmes-Weinstein monofilament (1.0 g) over the anterolateral shoulder of both shoulders and asking the patient to report if they could feel the stimulus.

### 2.3. Left/Right Judgement Task Procedure

**Figure 1:**
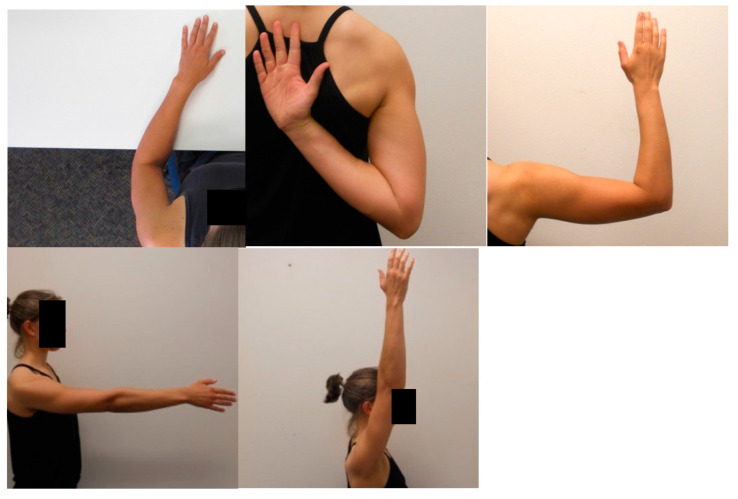
Examples of shoulder images displayed during Shoulder Left/Right Judgement Task.

The shoulder LRJT involved participants viewing a series of 40 shoulder images in random order on a computer screen and judging whether the observed images were of left or right shoulders. Five right shoulder joint images were developed to represent the shoulder in a variety of postures of increasing awkwardness or complexity: at 0° elevation, 90° flexion, 90° abduction and 90° external rotation, 180° flexion and hand behind back (Figure 1). These images were then digitally mirrored to produce five left shoulder images. Each of these ten images were then presented to the subject in four orientations: upright (0°), 90° clockwise, 90° anticlockwise, and inverted (180°). Participants sat comfortably in front of a computer screen and struck a computer key to indicate their choice as quickly and as accurately as possible. This shoulder LRJT was specifically developed for use in shoulder pain clinical groups and is valid and reliable, see Breckenridge et al. 2017 for further detail [58]. The task was delivered online using the software program Recognise™ (noigroup.com, Adelaide, Australia), described in detail previously [59,60] and shown to be reliable [61,62].

Mean response times (RT) in milliseconds and accuracy as a percentage of correct responses were reported as the two main outcome measures. Only RT for correct responses were used in the analysis. Responses of less than 500 ms were considered invalid as it is thought this is the minimum time required for identification of a pictured body part [63]. Responses that timed out i.e., reached 15,000 ms, indicate a failure to respond and were treated as incorrect responses.

### 2.4. Two-Point Tactile Discrimination Procedure

Two-point discrimination was assessed using a modified protocol from the method described by Moberg [64]. Stainless Steel Vernier Callipers with a digital readout and reported precision of +/−0.3 mm were used for measurement. The callipers were adapted with a pair of 4.08 Semmes-Weinstein monofilaments calibrated to deliver 1.0g of force (North Coast Medical, Gilroy, CA, USA) [65]. The callipers were applied vertically, parallel, at a 90° angle to the anterolateral aspect of the shoulder, the monofilaments touching the shoulder at precisely the same time and held stationary for one second.

Participants were instructed to say ‘one’ if they perceived a single point of contact, ‘two’ for two points and ‘don’t know’ if they were unsure. Participants were asked to indicate if they felt the callipers were applied asynchronously. Catch trials where only one point was applied to the skin were used to ensure subjects were not guessing. The testing procedure was based on that described by Wand [66], commencing with an ascending trial with the callipers initially set at 0 mm distance and increased by 10 mm increments until the participant reported two distinct points. Testing was repeated up to three times at each level to ensure consistency. This was followed by a descending trial starting well above the maximum level achieved during the ascending run, the monofilament calliper distance was reduced by 10mm increments until the participant reported one monofilament consistently. This procedure of ascending and descending trials was repeated twice. The mean of ascending trials and descending trials was calculated as the two-point discrimination threshold. This procedure was repeated for both the painful frozen shoulder and the contralateral healthy shoulder. 

### 2.5. Statistical Analysis

All data were analysed using IBM SPSS Statistics for Mac, Version 25.0 (IBM Corp., Armonk, NY, USA). Paired samples t-tests were used to compare LRJT mean RTs and accuracy scores for images corresponding to the frozen shoulder and the healthy shoulder. Data were transformed where not normally distributed. Pearson’s correlation was used to investigate any potential relationship between LRJT outcome measures and NRS pain intensity measures, disability score (SPADI) and reported duration of symptoms.

Two-point discrimination data were determined to be normally distributed as assessed by visual inspection of Normal Q-Q plots. Mean side to side differences in two-point discrimination data between the affected and unaffected shoulders were investigated with a paired sample t-test. Pearson’s correlation was used to investigate any potential relationship between side to side difference in two-point discrimination thresholds and NRS pain intensity measures, disability score (SPADI), reported duration of symptoms and age. 

## 3. Results

### 3.1. Participant Characteristics

Eighteen frozen shoulder participants (13 female) were recruited. Individual and mean (SD) demographic data are presented in Table 1. Participants were of middle age (mean 52.9 ± 8.1 years), had chronic symptoms (duration mean 22.0 ± 9.7 weeks), were suffering moderately severe pain: worst pain NRS mean 8.0/10 (±2.5); average pain NRS mean 5.0/10 (±2.3); and reported moderate levels of disability with a SPADI mean score of 59.2 (±20.4)%. Passive shoulder range of motion for flexion, external rotation and hand behind back are reported in Table 2. Mean passive flexion (93.9°) and external rotation (19.4°) of the affected shoulder were both clinically and statistically significantly reduced compared to the unaffected shoulder (flexion 172.5°; external rotation 70.1°) (*p* < 0.001). Hand behind back range was also substantially reduced on the affected side. Light touch skin sensory testing over the affected and unaffected shoulders revealed a normal sensory profile.

### 3.2. Left/Right Judgement

**Figure 2:**
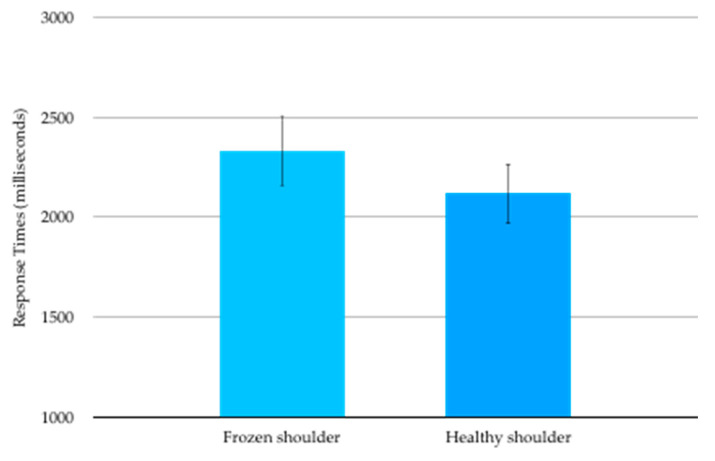
Shoulder Left/Right Judgement mean (SE) response times in milliseconds.

Mean (SD) RT for images corresponding to the affected shoulder was 2331(1576) ms and for the healthy shoulder was 2118 (1262) ms (Figure 2). The RT data were not normally distributed and were transformed to satisfy normality requirements prior to analysis. Mean RT for images corresponding to the affected shoulder was statistically significantly slower than mean RT for images corresponding to the healthy shoulder (t_17_ = 2.345, *p* = 0.031).

Mean accuracy on the LRJT corresponding to affected frozen shoulder was 87.8% (16.8) and for the healthy shoulder was 85.3% (17.4). The accuracy data were not normally distributed and were transformed to satisfy normality requirements prior to analysis. There was no side-to-side difference in LRJT accuracy (t_17_ = −0.625, *p* = 0.540).

There was no statistically significant correlation between LRJT mean RTs and worst pain scores (*p* = 0.747), average pain scores (*p* = 0.236), SPADI scores (*p* = 0.829) or the duration of pain (*p* = 0.353). Neither was there a statistically significant correlation between LRJT accuracy and worst pain scores (*p* = 0.704), average pain scores (*p* = 0.811), SPADI scores (*p* = 0.760), nor pain duration (*p* = 0.765).

### 3.3. Two-Point Discrimination

**Figure 3:**
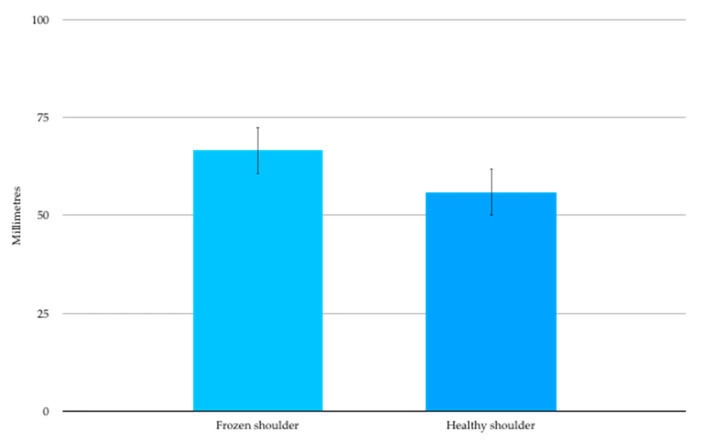
Two-point Discrimination mean (SE) thresholds in millimetres.

The data from one participant was considered invalid and thus excluded from the analysis as they were consistently caught guessing with catch trials. For the remaining 17 participants the mean two-point discrimination threshold (SD) was 66.6 (25.1) mm over the affected shoulder (Figure 3). This was significantly larger than the contralateral healthy shoulder 55.9(25.7) mm, (t_16_ = 4.847, *p* < 0.001) and represented a mean side to side difference of 10.7mm (95%CI 6.0 to 15.4mm).

There was no significant correlation between mean side to side difference in two-point discrimination threshold and average pain (*p* = 0.933), worst pain (*p* = 0.758), SPADI score (*p* = 0.574), duration of symptoms (*p* = 0.941) or age (*p* = 0.325). There were no significant correlations between LRJT outcome measures (RT and accuracy) and two-point discrimination (*p* ranges from 0.25–0.86).

## 4. Discussion

The primary aims of this study were to determine if motor imagery performance and/or tactile acuity were altered in people with unilateral frozen shoulder. We hypothesised that both LRJT performance and two-point discrimination would be altered in people with frozen shoulder, and both hypotheses were supported. This implicates the presence of cortical changes in people with the debilitating chronic shoulder pain condition termed frozen shoulder. 

People with frozen shoulder were slower at recognising images that corresponded to their frozen shoulder compared to images that corresponded to their healthy unaffected shoulder. Our results concur with those of Mena-del Horno et al. [42] and add to the findings of a recent motor imagery in chronic pain systematic review, which reported overall slower RTs of the affected body part in peripheral chronic musculoskeletal pain conditions [41]. One possible explanation of this poorer motor imagery performance is that the central processing related to implicit motor imagery is affected in people with frozen shoulder, which could reflect maladaptive neuroplastic change. The LRJT involves a three-step process: one makes an initial choice of left or right shoulder, then moves the virtual shoulder into a position to match that shown on the screen, and lastly one confirms/denies the match. If the match is incorrect the process is repeated with the contralateral virtual shoulder thus extending the response time. One explanation for the increased RTs demonstrated by people with frozen shoulder is that RTs vary according to the pain/stiffness that would be experienced if the actual painful shoulder was moved. If the virtual shoulder is moved cautiously into position RTs would be increased. Alternatively, people with frozen shoulder may make more erroneous initial choices but ultimately choose the correct response, resulting in longer response times. This poor implicit motor imagery performance suggests delays in neural processing and possible changes in somatosensory, motor related, or proprioceptive maps in people with frozen shoulder. However, as the LRJT is an indirect method of investigating cortical changes, more direct methods of investigation with neuroimaging and/or neurophysiological testing in people with frozen shoulder, are warranted to determine if this is the case [20]. 

Another explanation for the impaired motor imagery performance demonstrated in this study by people with frozen shoulder could be alterations in proprioception, whether a reduction in reliance on proprioceptive inputs, a shift in reliance from the sensorimotor to visuospatial inputs, or a change in proprioceptive weighting. An unwillingness to move the shoulder due to pain could reduce sensory and proprioceptive inputs forcing a reliance on visuospatial inputs such as occurs in spinal cord injured people [67]. Alternatively, a proprioceptive transition from the shoulder to another body area similar to that found in people with low back pain) [68] could affect LRJT performance. 

The RTs on the LRJT demonstrated by people with unilateral frozen shoulder in this study were considerably slower than those previously reported for healthy pain-free people [69] for both their frozen shoulder and healthy shoulders, indicating possible bilateral motor imagery deficits. Bilateral motor imagery impairments in unilateral musculoskeletal conditions implicates the involvement of central mechanisms [70]. While speculative, this finding of a bilateral reduction in LRJT RT suggests that bilateral cortical processing deficits and/or reorganisation are associated with frozen shoulder. Given that frozen shoulder has a relatively high recurrence rate on the contralateral side [2,71] the finding of bilateral motor imagery impairment in people with unilateral frozen shoulder is suggestive of a contributing role for cortical changes in the development of frozen shoulder. Further studies will need to be conducted to determine if there is a causal link between brain changes and frozen shoulder.

Recent neuroscience studies suggest a sequential organisation of imagined movement to complete a LRJT [72]. Participants imagine moving their limb from a palm down reference position (hands on table) to a supinated and rotated position, and this is reflected in the sequential activation of hand muscles then wrist supination. In that study mental rotation timing was associated with corticospinal excitability of muscles that would be activated during active movement execution. We would expect that corticospinal excitability would differ in people with frozen shoulder, compared to the pain free side, and to healthy controls, further implicating M1 changes in frozen shoulder. However, this assumption is speculative based on this evidence and would need to be explored in future neurostimulation studies.

Tactile acuity was altered in the people with frozen shoulder examined in this study with a notably elevated two-point discrimination threshold over the painful frozen shoulder compared to the healthy pain-free shoulder. Our findings agree with a recent similar study [42] and align with the results of a systematic review examining tactile acuity in people with chronic musculoskeletal pain [39] which reported diminished acuity in arthritis, complex regional pain syndrome and chronic low back pain. There was no difference in light touch sensation over the affected and unaffected shoulders in our frozen shoulder participants, so the two-point discrimination threshold changes are likely to be of central rather than peripheral origin. One explanation for this finding of altered tactile discrimination over the affected shoulder may be disruption of somatosensory representations in S1 of the affected shoulder, leading to tactile acuity imprecision. Evidence supporting this hypothesis of S1 cortical map disruption have been reported in complex regional pain syndrome [22] and chronic back pain [21], and further reinforced in that these changes appear to parallel two-point discrimination threshold impairments [73].

Shoulder region two-point discrimination thresholds in healthy people reportedly range from 31 mm to 44 mm [65,74,75,76] and these normative values are considerably smaller than the average value of 55.9 mm found over the non-painful shoulder in the frozen shoulder cohort in this study. Tactile discrimination thresholds are known to increase with age [77,78], and this difference may reflect the fact that these normative values were derived from younger samples (mostly university aged 20–46 years) than the frozen shoulder cohort reported in the current study (mean age of 52.8 years). On the other hand, frozen shoulder is reported to have a relatively high recurrence rate of 8–17% [2,71] in the contralateral shoulder. If this higher two-point discrimination threshold in the non-painful shoulder in the current frozen shoulder cohort is reflective of contralateral cortical somatosensory changes, it may provide support for a contributing role of central changes in the development of frozen shoulder.

We did not find a relationship between performance on the shoulder LRJT or two-point discrimination acuity, and either reported pain and disability scores or the duration of symptoms. This finding is consistent with a recent systematic review which found little evidence overall of a relationship between tactile acuity and pain intensity and duration [39] in chronic pain conditions including complex regional pain syndrome, chronic low back pain and osteoarthritis of the knee.

Does ongoing shoulder pain and stiffness affect proprioception and result in poorer motor imagery performance, or does altered proprioceptive and other afferent signalling [79] contribute to or perpetuate chronic shoulder pain? In the first instance shoulder pain would precede motor imagery deficits, in the latter motor imagery deficits would come prior to shoulder pain. The cross-sectional design used in this study does not allow a conclusion to be made about whether poorer motor imagery performance, reduced tactile acuity and possible associated neuroplastic changes are a result of frozen shoulder or, conversely, a contributing factor to the development of frozen shoulder. To further explore this question the shoulder LRJT and two-point discrimination tests could be used to monitor motor imagery performance and tactile acuity longitudinally in a clinically confirmed frozen shoulder sample to determine if and when alterations become apparent after the development of frozen shoulder, if any alterations resolve with time and if they are associated with development of symptoms in the initially unaffected shoulder. If applied during the rehabilitation time period, either test may prove to be a valuable tool to evaluate the impact of rehabilitation on motor imagery performance, tactile acuity related cortical changes and to gain insight into the aetiology of frozen shoulder.

The findings of this study that maladaptive neuroplastic changes are associated with frozen shoulder suggest that treatment aimed at reversing these changes are warranted. Treatment options like graded motor imagery that aims to restore proprioceptive and motor cortical networks, and sensory discrimination training that focuses on somatosensory change, or a combined approach, may be appropriate management alternatives for people with frozen shoulder.

There are several limitations to this study to address in future research. Internal validity was potentially threatened because assessors were not blind to which was the affected shoulder and there was no healthy control group with which to compare results. Two-point discrimination testing has received criticism regarding sensitivity, reliability and assessor bias [80,81]. However, we sought to ensure best practice and good reliability by: having one researcher perform all testing [82]; by describing the experimental testing procedure in detail [80]. A novel strength of this study was to precisely control the calliper application force by the use of force calibrated monofilaments [65,80]. Nevertheless, replication of this work with assessor blinding is warranted. It is possible that there were subtle interactions between frozen shoulder disease and motor imagery performance that were missed in our study. We therefore suggest that future studies are appropriately planned and are powered to enable detailed analysis of interactions between parameters of the shoulder LRJT e.g., orientation and awkwardness/complexity of shoulder image.

## 5. Conclusions

We demonstrated altered motor imagery performance and tactile acuity thresholds in people with frozen shoulder. One explanation for these findings is altered sensorimotor processing and/or disrupted sensorimotor cortex representations of the affected shoulder. These findings indicate that frozen shoulder may be accompanied by maladaptive neuroplastic changes, and therefore a case exists for the use of treatment aimed at reversing these changes to improve clinical outcomes for people with chronic shoulder pain.

## Figures and Tables

**Table 1:** Participant demographic data.

Participant	Affected Shoulder	Age Years	Gender	Duration Frozen Shoulder Weeks	Worst Pain NRS /10	Average Pain NRS /10	SPADI Score %
1	Left	69	Female	24	8	5	55
2	Right	48	Female	9	9	5	66
3	Left	45	Male	13	10	7	67
4	Left	57	Male	13	9	5	46
5	Left	45	Male	13	10	5	24
6	Left	50	Female	32	10	9	88
7	Left	50	Male	20	10	6	62
8	Left	52	Female	13	8	5	54
9	Right	41	Female	36	6	3	35
10	Left	67	Female	28	0	0	17
11	Right	58	Male	20	9	8	79
12	Left	46	Female	20	8	6	68
13	Left	63	Female	28	10	5	87
14	Left	57	Female	11	7	2	59
15	Right	43	Female	26	10	7	74
16	Right	49	Female	44	9	7	76
17	Right	59	Female	30	6	3	39
18	Left	53	Female	16	5	2	70
Mean (SD)		52.9(8.1)		22.0(9.7)	8.0(2.5)	5.0(2.3)	59.2(20.4)

**Table 2:** Passive range of motion in degrees for affected frozen shoulder and healthy contralateral shoulder with means and standard deviation for flexion and external rotation.

Passive Range of Motion						
Affected Shoulder				Healthy Shoulder		
Participant	Flexion	Ext. Rot.	HBB.	Flexion	Ext. Rot.	HBB
1	90	15	-	160	55	-
2	85	-5	hip	175	75	T8
3	105	5	butt	180	45	T6
4	105	15	SIJ	180	60	T7
5	100	55	L1	175	70	T7
6	45	-10	butt	180	70	T6
7	105	10	hip	170	70	T7
8	100	20	L5	180	75	T4
9	115	55	SIJ	160	70	T10
10	95	50	L5	160	70	T10
11	100	25	S1	180	70	T10
12	90	10	-	180	90	-
13	90	30	hip	180	80	T10
14	90	15	butt	185	95	T6
15	105	35	butt	160	80	T10
16	90	5	-	150	35	-
17	85	10	-	175	80	-
18	95	10	butt	180	80	T6
Mean (SD)	93.9(14.7)	19.4(19.0)		172.5(10.0)	70.6(14.8)	

For hand behind back (HBB) measurement was thumb tip to landmark e.g., hip, buttock(butt), L1 spinous process, T4 spinous process, SIJ sacroiliac joint. “-” = not recorded.
